# Broadening the Biocatalytic Toolbox—Screening and Expression of New Unspecific Peroxygenases

**DOI:** 10.3390/antiox11020223

**Published:** 2022-01-24

**Authors:** Sebastian Bormann, Harald Kellner, Johanna Hermes, Robert Herzog, René Ullrich, Christiane Liers, Roland Ulber, Martin Hofrichter, Dirk Holtmann

**Affiliations:** 1 Industrial Biotechnology, DECHEMA Research Institute, Theodor-Heuss-Allee 25, 60486 Frankfurt am Main, Germany; sb.bormann@googlemail.com (S.B.); hermes_johanna@web.de (J.H.); 2 International Institute Zittau, Technical University of Dresden, Markt 23, 02763 Zittau, Germany; harald.kellner@tu-dresden.de (H.K.); robert.herzog1@tu-dresden.de (R.H.); rene.ullrich@tu-dresden.de (R.U.); christiane.liers@tu-dresden.de (C.L.); martin.hofrichter@tu-dresden.de (M.H.); 3 Institute of Bioprocess Engineering, University of Kaiserslautern, Gottlieb-Daimler-Strasse 49, 67663 Kaiserslautern, Germany; ulber@mv.uni-kl.de; 4 Institute of Bioprocess Engineering and Pharmaceutical Technology, Technische Hochschule Mittelhessen, Wiesenstraße 14, 35390 Giessen, Germany; 5 Research Division Bioresources, Fraunhofer Institute for Molecular Biology and Applied Ecology (IME), Ohlebergsweg 12, 35392 Giessen, Germany

**Keywords:** unspecific peroxygenases, EC 1.11.2.1, *Pichia pastoris*, direct expression

## Abstract

Unspecific peroxygenases (UPOs) catalyze the selective transfer of single oxygen atoms from peroxides to a broad range of substrates such as un-activated hydrocarbons. Since specific oxyfunctionalizations are among the most-desired reactions in synthetic chemistry, UPOs are of high industrial interest. To broaden the number of available enzymes, computational and experimental methods were combined in this study. After a comparative alignment and homology modelling, the enzymes were expressed directly in *P. pastoris.* Out of ten initially selected sequences, three enzymes (one from *Aspergillus niger* and two from *Candolleomyces aberdarensis*) were actively expressed. Cultivation of respective expression clones in a bioreactor led to production titers of up to 300 mg L^−1^. Enzymes were purified to near homogeneity and characterized regarding their specific activities and pH-optima for typical UPO substrates. This work demonstrated that directed evolution is not necessarily required to produce UPOs in *P. pastoris* at respective titers. The heterologous producibility of these three UPOs will expand the toolbox of available enzymes and help to advance their synthetic application.

## 1. Introduction

Unspecific peroxygenases (UPOs; EC 1.11.2.1), members of the heme-thiolate proteins, catalyze the selective transfer of single oxygen atoms from peroxides (H_2_O_2_, ROOH) to diverse target molecules including un-activated hydrocarbons [1,2]. In general, hydrogen peroxide driven biocatalysis combines the high oxidation power of H_2_O_2_ and its environmentally friendly properties with the high selectivity and further advantages of enzymatic oxidation reactions [3]. UPOs are found with few exceptions (i.e., *Peronosporomycetes*) exclusively in true fungi (Eumycota). Although their exact physiological function is unknown, they have been widely studied regarding their application potential, since specific oxyfunctionalizations are among the most-desired reactions in synthetic chemistry [4]. The easiness and the wide range of reactions catalysed by peroxygenases make them promising catalysts for preparative oxy-functionalizations [5].

Nowadays, thousands of UPO sequences can be found in genome-sequenced fungi, but only a handful of wild-type enzymes and a few recombinantly expressed UPOs have been characterized and used in lab-scale studies. Examples are wild-type UPOs from *Cyclocybe (Agrocybe) aegerita*, *Marasmius rotula*, or *Chaetomium globosum*, recombinantly produced UPOs from *Coprinopsis cinerea*, *Collariella (Chaetomium) virescens*, *Daldinia caldariorum*, *Hypoxylon* sp. EC38, or evolved variants from *Candolleomyces* (*Psathyrella*) *aberdarensis* and *Cyclocybe aegerita* [6,7,8,9]. Chloroperoxidase (CPO; EC 1.11.1.) from the ascomycetous sooty mold *Leptoxyphium (Caldariomyces) fumago* forms an exception, since it phylogenetically belongs to the UPOs (but not according to its EC classification) and can be produced as a wild-type enzyme in larger amounts (>500 mg L^−1^).

One major bottleneck so far remains the lack of recombinant expression of a representative selection of UPO genes with satisfactory expression levels (>100 mg L^−1^). In particular, the common *Escherichia coli* expression system has not properly worked for many years and still does not when it comes to UPOs. Only recently has it become possible to produce small amounts of active UPOs using a modified *E. coli* expression system, although this did not work in the case of all UPO constructs tested and always occurred without glycosylation [7]. In comparison, yeast systems (*Saccharomyces cerevisiae* and *Pichia pastoris* (i.e., *Komagataella phaffii*) appear to be more promising for recombinant expression and specifically for enzyme evolution. Examples for the successful expression and evolution are the UPOs from *Cyclocybe aegerita* and *Candolleomyces aberdarensis* [9]. However, prior to the successful expression of appreciable rUPO titers in *P. pastoris*, these corresponding genes had to be evolved, particularly in the signal peptide and sometimes also in the core domain [6].

In this study, we took another approach by selecting candidates after comparative alignments and homology modelling, and then proceeding with their direct expression in *P. pastoris*, thereby bypassing the time-consuming step of enzyme evolution in *S. cerevisiae*. With this approach, we aimed to accelerate the search for functional active UPO enzymes, which could provide a suitable platform for subsequent manipulations. In total, we tested 10 synthetically prepared and codon-optimized UPO genes for recombinant expression. The putative UPO genes (Table 1) were identified based on sequence motives, and a subset of protein sequences was investigated in this work. The selection was biased towards “short” UPOs (<30 kDa) as their expression in yeast has remained elusive, while the well-expressible UPOs from the *Cyclocybe (Agrocybe) aegerita* variant PaDa-I (*Aae*UPO-PaDa-I) and *Coprinopsis cinerea* (rCciUPO) belong to the “long” UPO subset [10].

## 2. Material and Methods

### 2.1. UPO Candidate Selection

The overall selection followed an UPO database and alignments including more than 2000 protein sequences used in Hofrichter et al. [1]. Especially sequences larger than 500 amino acids and shorter than 200 amino acids as well as partial sequences were excluded. Sequences with problems derived from wrong intron-exon boundaries of the predicted genes during genome sequencing projects were removed, as well as sequences with missing core motifs like the PCP and EHD-S/EGD-S motifs distal and proximal of the heme center [1]. Then, 10 candidates displaying the diverse taxonomical and biochemical nature of UPOs (i.e., short and long UPOs from Ascomycota and Basidiomycota) were selected after tertiary structure modelling using I-TASSER [11] or C-I-TASSER [12] and visualization in PyMOL 2 (Schrödinger, New York, NY, USA). Thereby, especially the variable substrate channel architecture was in the focus for selection. For docking simulations of the substrate veratryl alcohol, the PyMOL plugin NRGSuite (version 2.48I, Dr. Rafael Najmanovich in Université de Sherbrooke, Sherbrooke, Quebec, Canada) was used [13]; a small phylogeny of the selected candidates and important UPO references was calculated using maximum likelihood (PhyML 3, substitution model: LG + I + G, [14] implemented in Geneious Prime 2020 [15]). Signal peptide prediction was carried out using SignalP 5 [16].

### 2.2. Chemicals, Strains and Plasmids

Chemicals were purchased from either Sigma-Aldrich (Schnelldorf, Germany), Carl-Roth (Karlsruhe, Germany), or VWR (Darmstadt, Germany) in >97% purity. *Pichia pastoris* X33 and zeocin were purchased from ThermoFisher (Dreieich, Germany). Competent *Escherichia coli* NEB 5-alpha cells were purchased from New England Biolabs (NEB, Frankfurt, Germany). Expression plasmid pPpB1 was provided by the group of Anton Glieder (Technical university Graz, Graz, Austria).

### 2.3. Preparation and Transformation of Expression Plasmids

Genes of putative UPO genes were synthesized by BioCat (Heidelberg, Germany) in pUC19. Coding sequences (CDSs) are given in the Supplementary Information (SI). Genes and the plasmid backbone pPpB1 were amplified by polymerase chain reaction (PCR) using Q5 polymerase (NEB) with the following protocol: initial denaturation at 98 °C for 30 s, followed by 30 cycles with 10 s at 98 °C, 15 s at primer melting temperature (T*_M_*, SI Table S1, 30 s kb^−1^ at 72 °C, and a final extension at 72 °C for 120 s. PCR products were cleaned up using the DNA clean and concentrator-5 kit (Zymo Research, Freiburg, Germany). Amplified genes and plasmid backbone were mixed in 3:1 molar ratio, digested by *Dpn*I (NEB), ligated without further purification using isothermal assembly [17], and transformed into *E. coli* NEB 5-alpha according to the manufacturer’s protocol for heat shock transformation. *E. coli* NEB 5-alpha colonies were selected on low-salt LB (10 g L^−1^ tryptone, 5 g L^−1^ yeast extract, 5 g L^−1^ NaCl) agar (15 g L^−1^) containing 25 mg L^−1^ zeocin. Plasmids were purified from cultures after overnight cultivation in low-salt LB + zeocin at 37 °C and 180 rpm using the GeneJET Plasmid MiniPrep Kit (ThermoFisher, Dreieich, Germany). Sequences of the cloned UPO-genes were verified by Sanger sequencing (Eurofins, Hamburg, Germany). Plasmids were linearized by digestion with *Pme*I and cleaned up (vide supra). Competent *P. pastoris* X33 cells were prepared as follows: 500 mL of YPD.media (10 g L^−1^ yeast extract, 20 g L^−1^ peptone, 20 g L^−1^ glucose) was incubated inoculated from an overnight culture and grown overnight to an optical density determined at 600 nm (OD_600_) of 1–1.5. Cells were pelleted at 1500 g, washed twice with 250 mL ice-cold water, washed once with 20 mL ice-cold 1 M sorbitol, and finally resuspended in 1 mL 1 M sorbitol. Linearized plasmid (4–8 µg) and 80 µL competent cells were mixed in a pre-cooled 2 mm electroporation cuvette and incubated for 5 min. Electroporation was carried out using a Gene Pulser Xcell (Bio-Rad, Dreieich, Germany) with settings for *Saccharomyces cerevisiae*. Immediately after electroporation, 1 M sorbitol was added and cells were transferred to 1.5 mL reaction vials and incubated at 30 °C for about 60 min, after which an equal volume of YPD was added and incubation was continued for 30–60 min. Aliquots were spread onto YPD agar containing 1 M sorbitol and 100 mg L^−1^ zeocin and incubated at 30 °C for several days until colonies were visible.

### 2.4. Screening of P. pastoris Clones for UPO Activity

*P. pastoris* X33 colonies (24 colonies for each UPO-expression plasmids) from zeocin selection plates were picked into 96 deep-well plates (DWPs) and cultivated at 30 °C, 800 rpm, >80% humidity in a Microtron (InforsHT) according to the screening strategy described by [18] with the following changes. The initial addition of methanol-containing medium was carried out when glucose was depleted as determined by sampling of several wells using a Reflecoquant^®^ (Merck, Darmstadt, Germany) system. Following the initial methanol-addition, additional methanol was fed every 17–24 h. Samples for activity determination (50 µL) were taken before feeding of methanol. Cultivation was terminated after about 100 h. Enzyme activity in the culture supernatant was determined using the 2,2′-Azino-*bis*(3-ethylbenzthiazoline-6-sulfonic acid) diammonium salt (ABTS) and 5-Nitro-1,3-benzodioxole (NBD) assays (vide infra) [19].

### 2.5. Expression of Recombinant Protein

In order to produce the proteins, those *P. pastoris* X33 clones that exhibited UPO activity in the screening were cultivated in a 1-L (working volume) 4× DASGIP parallel bioreactor system (Eppendorf). Cultivations were carried out as described by Cino et al. 1999 [20] with the following adaptions. Reactors were inoculated to an OD_600_ of 1–2 at an initial cultivation volume of 500 mL (50% working volume). Glycerol and methanol feed rates during the respective fed-batch phases were based on the dissolved oxygen (DO) concentration which had a set-point of 30% and was controlled by stirring rate (≤1200 rpm), aeration rate (≤50 L min^−1^), and oxygen content (21–100%); pH was held above 5 by the addition of 15% ammonia. Temperature was set at 30 °C. DO-based feeding rates for glycerol were 0–20% DO, 2–4 mL h^−1^, 20–30% DO, 4–8 mL h^−1^, 30–50% DO, 8–10 mL h^−1^, >50% DO, and 10 mL h^−1^. For methanol, the profile was: 0–25% DO, 0–3 mL h^−1^, 25–30% DO, 3–4 mL h^−1^, 30–40% DO, 4–6 mL h^−1^, >40% DO, and 6 mL h^−1^.

### 2.6. Protein Purification

Culture broth was removed from bioreactors and initially clarified by centrifugation at 15,000× *g*. Further clarification was carried out by crossflow-filtration using a 0.2 µm membrane cassette (Vivaflow 200 0.2 µm PES, Sartorius, Göttingen, Germany). High-molecular weight compounds were concentrated using a 10 kDa cut-off crossflow-ultrafiltration membrane module (Vivaflow 200, 10 kDa, PES, Sartorius, Göttingen, Germany) followed by a further concentration using ultrafiltration centrifugal concentrators (Vivaspin 20 mL, 3 kDa, Sartorius, Göttingen, Germany). Fast protein liquid chromatography (FPLC) was carried out using an Azura FPLC system (Knauer, Berlin, Germany). Concentrated samples were clarified using 0.2 µm PES syringe filters (VWR, Darmstadt, Germany) and mixed with an equal volume of 10 mM potassium phosphate buffer (KP*i*) pH 7 with 2 M ammonium sulfate prior to hydrophobic interaction chromatography (HIC). HIC was carried out using a 250 mL (3.6 cm inner diameter) column packed with Toyopearl butyl 650 M (Tosoh, Griesheim Germany). Samples were applied directly to the column at a flow-rate of 4 mL min^−1^. Proteins were eluted using a binary gradient with buffers consisting of 10 mM KP*i* pH 7 with 1 M ammonium sulfate (buffer A) and without ammonium sulfate (buffer B) at a flow rate of 8 mL min^−1^. The elution profile was 1 column volume (CV) buffer A followed by a linear gradient of 100% buffer A to 100% buffer B over 1.4 CV. Fractions with a sufficiently high Reinheitszahl (Absorption*_Soret_*/Absorption_280nm_) (R*Z*) were collected, concentrated, and dialyzed against size exclusion chromatography (SEC) buffer consisting of 10 mM KP*i* pH 7 with 150 mM NaCl. SEC was carried out using a HiPrep 16/60 Sephacryl S-300 HR column (1.6 cm inner diameter, 120 mL CV, GE Healthcare, Solingen, Germany). Samples were applied to the column using a sample loop (1.1 mL) at a flow-rate of 0.5 mL min^−1^, which was kept constant for 0.7 CV and increased to 1 mL min^−1^ afterwards. Samples with a sufficiently high R*Z* were collected, concentrated, and dialyzed against 100 mM KP*i* pH 7 for characterization experiments.

### 2.7. Assays

Enzyme activity assays were carried out using a UV-1700 (Shimadzu, Duisburg, Germany) in semi-micro poly(methyl methacrylate) (PMMA) cuvettes (pathlength 1 cm) at room temperature. For screening of *P. pastoris* X33 clones, assays were carried out in 96-well flat bottom microtiter plates (MTPs) using an Infinite 200 Pro (Tecan, Crailsheim, Germany). Assay volume in MTPs was 0.2 mL with the same composition as given for assays carried out in semi-micro cuvettes.

Routine ABTS (2,2′-azino-bis(3-ethylbenzothiazoline-6-sulfonic acid) assay consisted of 750 µL 100 mM Na_2_HPO_4_/citric acid buffer pH 4.4, 100 µL 3 mM ABTS, 50 µL 40 mM H_2_O_2_, and 100 µL sample. For the determination of pH-optima, 750 µL McIlvaine buffer (200 mM Na_2_HPO_4_/100 mM citric acid) was used. Absorption was determined at 420 nm; product concentration was calculated with an extinction coefficient of 36,000 M^−1^ cm^−1^ [21].

The pH optima of unspecific peroxygenases (UPOs) for veratryl alcohol (VA) were determined with the following assay mixture: 840 µL McIlvaine buffer, 100 µL 50 mM VA, 50 µL 40 mM H_2_O_2_, and 10 µL sample. Absorption was determined at 310 nm; product concentration (veratraldehyde) was calculated with an extinction coefficient of 9300 M^−1^ cm^−1^ [21].

The routine NBD assay (following demethylenation) consisted of 550 µL 100 mM KP*i* pH 7, 100 µL 5 mM NBD (5-nitro-1,3-benzodioxole) in acetonitrile, 200 µL H_2_O, 50 µL 20 mM H_2_O_2_, and 100 µL sample. pH optima of the UPOs for NBD were determined with the following assay composition: 750 µL McIlvaine buffer, 100 µL 5 mM NBD in acetonitrile, 50 µL 20 mM H_2_O_2_, and 100 µL sample. Absorption was determined at 425 nm; product concentration (4-nitrocatechol) was calculated with an extinction coefficient of 9700 M^−1^ cm^−1^ [19].

### 2.8. Protein Characterization

Protein concentrations were determined using the Bio-Rad Protein Assay Kit II (Bio-Rad, Dreieich, Germany) according to the manufacturer’s protocol using a bovine serum albumin dilution series for calibration. Sodium dodecyl sulfate–polyacrylamide gel electrophoresis (SDS-PAGE) was carried out using Mini-PROTEAN TGX precast gels and Laemmli sample buffer (Bio-Rad, Dreieich, Germany) with β-mercaptoethanol as a reducing agent, according to the manufacturer’s recommendations. Gels were stained with colloidal coomassie stain [22]. For glycosylation analysis, proteins were treated with PNGase F (NEB) according to the manufacturer-provided denaturing conditions protocol. Absorption spectra of purified proteins were determined on a NanoDrop 2000 (ThermoFisher, Dreieich, Germany).

## 3. Results and Discussion

### 3.1. Selection of Genes

**Figure 1:**
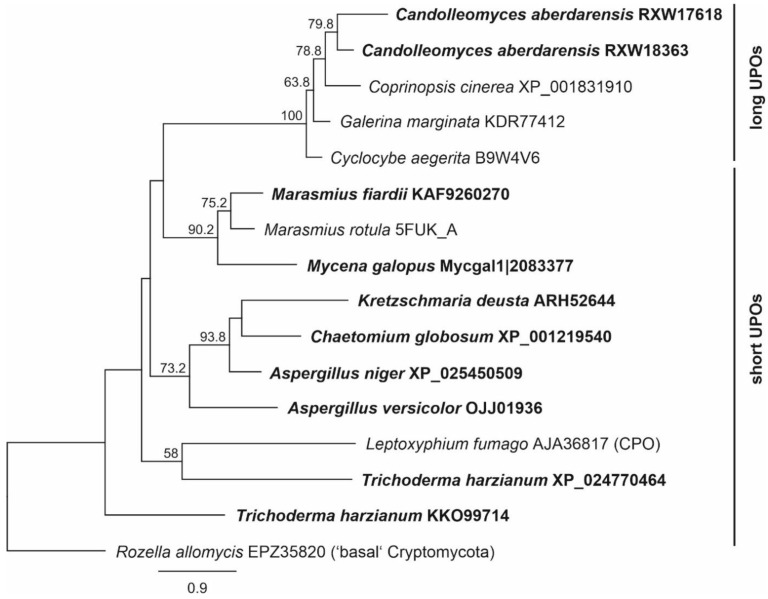
Maximum likelihood tree including unspecific peroxygenase (UPO) candidates for recombinant expression and references. Branch support was estimated by 500 bootstrap replicates.

**Figure 2:**
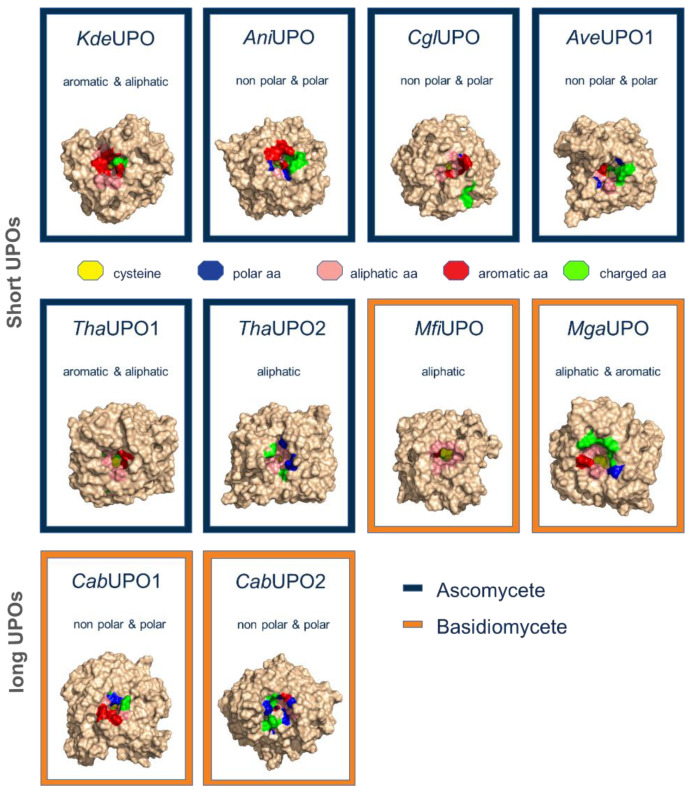
Homology modeled UPO candidates for recombinant expression.

Currently, several thousands of UPO genes are available in databases (NCBI, but also at JGI Mycocosm). As a first premise, we decided to select genes phylogenetically close to known and “working” reference UPOs, like those of the enzymes from *Cyclocybe aegerita* and *Marasmius rotula* (Figure 1). Secondly, the selected genes should include correct intron-exon boundaries (which was confirmed by extensive evaluation of alignments), a predicted signal peptide (e.g., using SignalP 5), and a substrate channel after homology modelling with different characteristics like more aliphatic or more aromatic amino acids (Figure 2). Phylogenetically, UPOs are highly diverse and include several (sub)families and clusters, like short and long UPOs as here and previously shown (Figure 1, [1]), and will provide plentiful candidates in future expression experiments.

### 3.2. Expression of Putative UPO Genes in P. pastoris

Putative UPO genes were cloned into the *Pichia pastoris* expression vector pPpB1 [23] under control of the methanol-inducible *aox1*-promoter, which has been successfully utilized to express *Aae*UPO-PaDa-I [24]. *P. pastoris* transformants were successfully generated for all 10 expression constructs. After transformation, 24 clones per construct were picked and cultivated in deep-well plates (DWPs) for 94 h. Supernatant from clones was tested for UPO activity using the ABTS and NBD assays. Clones transformed with expression plasmids for two UPOs from *Candolleomyces aberdarensis* (*Cab*UPO1 and *Cab*UPO2) and UPO from *Aspergillus niger* (r*Ani*UPO) exhibited activity with both assays, while no activity could be determined for either assay for all other constructs (Figure S1).

### 3.3. Protein Production in P. pastoris

**Figure 3:**
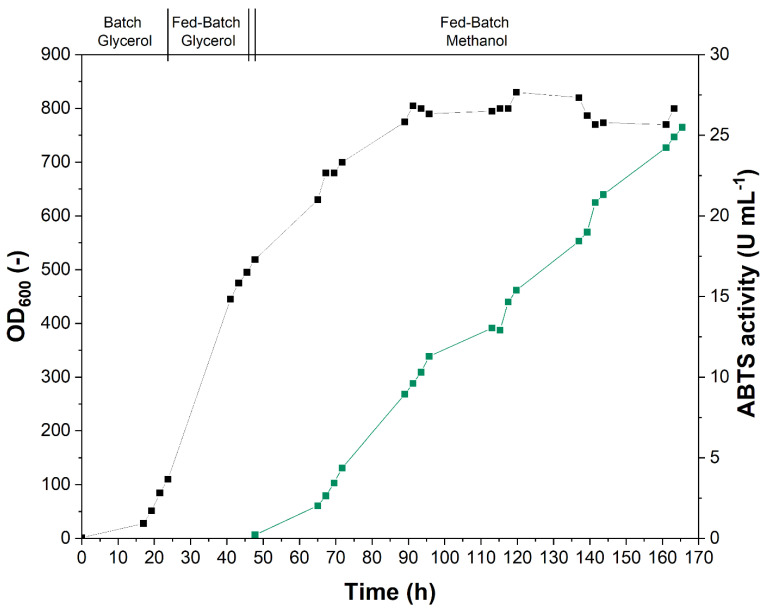
Production of recombinant r*Cab*UPO 2 with *P. pastoris* X33 in a 1-L bioreactor. Phases of the glycerol and methanol (fed)-batch process are indicated. A double bar marks the starvation phase preceding the methanol fed-batch. Cultivation was monitored by measurement of OD_600_ (∎) and enzyme production by determination of the UPO activity towards ABTS (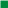).

The best-performing clones identified in the screening, i.e., those that exhibited the highest activities towards ABTS and NBD, were cultivated in a 1-L bioreactor in order to produce sufficient amounts of enzyme for subsequent purification and characterization. Fermentation of *P. pastoris* was carried out according to the widely used multistage fermentation protocol for methanol-induced protein production [20], which consists of a batch glycerol phase for initial biomass production, a glycerol fed-batch phase to accumulate biomass, and the main fed-batch methanol phase, in which only minor biomass accumulation occurs for the actual production of the protein of interest. Protein production, shown by the increase in ABTS activity after methanol induction, was successful for all three constructs identified in the screening (*Cab*UPO 1, *Cab*UPO 2, and r*Ani*UPO), as exemplarily shown in Figure 3 for r*Cab*UPO 2. Activity profiles of all cultivations are given in Figure S2.

Cultivations had to be terminated when the culture volume exceeded the reactors’ working volume due to continuous feeding of substrate. Judging from the protein production pattern exhibited, it might well be possible to further increase total protein titers with longer cultivation times. The volumetric ABTS activity that could be produced was in the same order of magnitude for all three proteins and about an order of magnitude lower than that of routine fermentations of recombinant UPO from *Cyclocybe aegerita* variant PaDaI (r*Aae*UPO-PaDa-I) [24]. In order to determine whether these differences were the result of lower secretion levels or of differences in specific enzyme activities, the cultivation broth was harvested for subsequent protein purification.

### 3.4. Protein Purification

**Figure 4:**
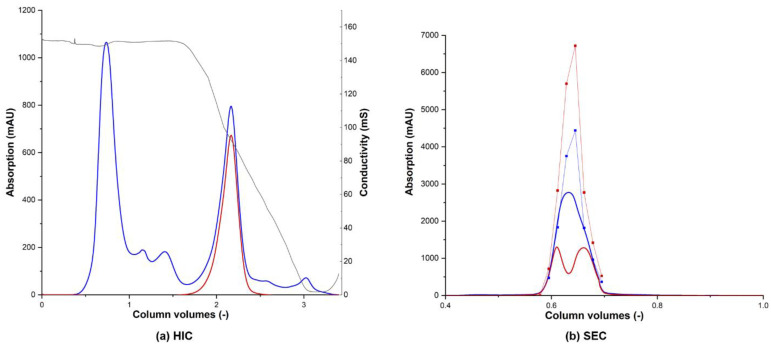
Purification of r*Cab*UPO 2 by hydrophobic interaction chromatography (HIC) (**a**) and size-exclusion chromatography (SEC) (**b**). Conductivity —, absorption was determined online (280 nm —, 420 nm —) and offline (280 nm 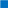, 420 nm 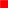).

Culture broth containing r*Cab*UPO1, r*Cab*UPO 2, and r*Ani*UPO was first clarified by centrifugation, then by 0.2 µm cross-flow filtration, and was afterwards concentrated via cross-flow ultrafiltration using a 10 kDa cut-off membrane before chromatographic purification by FPLC. The concentrated culture broth was first subjected to hydrophobic interaction chromatography (HIC). r*Cab*UPO 2 eluted in a single peak (Figure 4a), which was significantly narrower than that of r*Ani*UPO (Figure S3c), while r*Cab*UPO 1 eluted in two distinct peaks (Figure S3a). Analysis by SDS-PAGE showed that the two peaks of r*Cab*UPO 1 were of equal molecular weight (Figure S4). Since the first peak represented the majority of the enzyme, all further work was carried out using these fractions. r*Cab*UPO 2 was already purified to near homogeneity after HIC, while r*Cab*UPO 1 showed minor impurities around 35 kDa and r*Ani*UPO did not exhibit a major (distinct) protein band. Size exclusion chromatography (SEC) was carried out after concentration and dialysis of the pooled fractions from the HIC step to further purify the enzymes. While this was not strictly necessary for r*Cab*UPO 2, it was deemed appropriate to treat all enzymes similarly before biochemical characterization. While SEC for r*Cab*UPO 2 (Figure 4b) and r*Cab*UPO 1 (Figure S5a) showed few foreign proteins, as indicated by the overlap in heme-specific absorption and protein absorption, r*Ani*UPO could be partially separated from contaminating proteins (Figure S5c). Offline photometric measurement of the fractions collected showed that the double peak present in the chromatograms of r*Cab*UPO 2 and r*Cab*UPO 1 was an artifact and did not represent two distinct protein species. Surprisingly, r*Ani*UPO eluted earlier then r*Cab*UPO 1 and r*Cab*UPO 2, although its molecular weight based on the protein sequence was about 10 kDa lower.

The purification data are summarized in Table 2. The relatively low increases in specific activity, which were less than two-fold for all enzymes, indicated that the *P. pastoris* X33 culture broth contained only minor amounts of extracellular host-cell proteins. Based on the specific activity values determined after purification, maximum protein titers of 300 mg L^−1^ for r*Cab*UPO 1, 275 mg L^−1^ for r*Cab*UPO 2, and 140 mg L^−1^ for r*Ani*UPO were calculated for the bioreactor cultivations. These titers are similar to those of routine fermentations of r*Aae*UPO-PaDa-I with *P. pastoris* (about 200 mg L^−1^ in [24]).

### 3.5. Physicochemical Characterization

**Figure 5:**
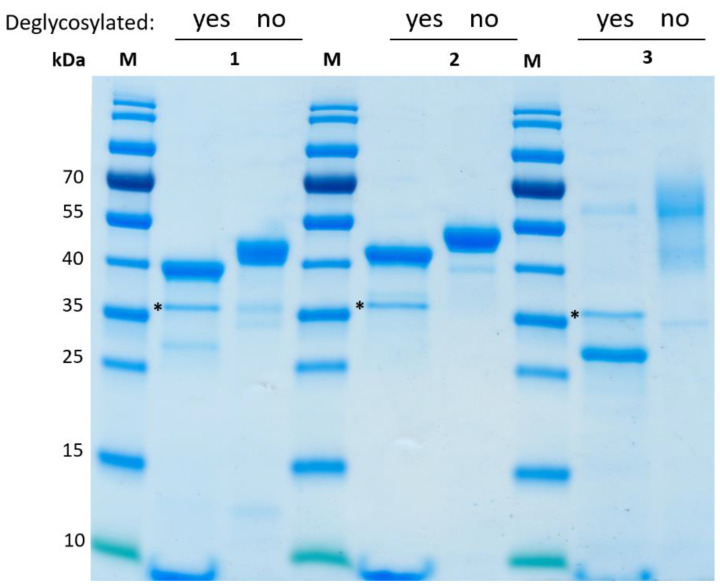
Analysis of protein purity and glycosylation content of purified proteins before and after N-deglycosylation with PNGase F. Lanes: M PageRuler Prestained (ThermoFisher), 1 r*Cab*UPO 1, 2 r*Cab*UPO 2, 3 r*Ani*UPO. The band representing PNGase F (35.9 kDa) is indicated (*).

Purified, concentrated, and dialyzed proteins were characterized concerning final purity and molecular weight by SDS-PAGE (Figure 5). r*Cab*UPO 1 and r*Cab*UPO 2 bands were in range of predicted weight, while r*Ani*UPO appeared as a non-distinct band at around 55 kDa and 40 kDa, which is significantly over the predicted weight of 27.6 kDa for the processed protein (see Table 1). *P. pastoris* has been reported to over-glycosylate foreign proteins, which was shown for various enzymes including a UPO [24]. Since high-mannose N-glycosylation is most prevalent in *P. pastoris* [25], proteins were deglycosylated using PNGase F in order to remove possible oligosaccharides. Deglycosylation led to a minor shift in molecular weight for r*Cab*UPO 1 and r*Cab*UPO 2 to slightly below and above 40 kDa, respectively. This is in good agreement with their predicted molecular weights of 38.3 kDa and 39.3 kDa (Table 1). Both major and minor bands of r*Ani*UPO shifted to a single band above 25 kDa after deglycosylation, which matches the predicted molecular weight of 27.6 kDa. This led us conclude that r*Ani*UPO becomes highly over-glycosylated when expressed in *P. pastoris* with an estimated glycosylation degree of up to 50%. This in turn results in a high degree of glycosylation-derived heterogeneity, as indicated by the presence of the hyper-glycosylated major and the less glycosylated minor bands. As such, differently glycosylated protein fractions were also observed for wild-type *Aae*UPO [2,26]. The results for all three rUPOs are in agreement with predicted N-glycosylation sites, and thereby the sequence of r*Ani*UPO having six potential N-glycosylation sites, whereas both sequences of *Candolleomyces* rUPOs showed only two to three sites.

Spectra of the purified enzymes exhibited the heme-thiolate typical Soret region around 420 nm, as well as the charge transfer bands at about 530 nm and 570 nm (Figure S6). The Reinheitszahl (RZ, Absorption*_Soret_*/Absorption_280nm_), which is often used as an indicator for the purity of heme-containing enzymes, was well above two for r*Cab*UPO 1, in accordance with the high degree of purity determined by SDS-PAGE. While r*Cab*UPO 2 was similarly pure by SDS-PAGE, its R*Z* was notably lower, which could have been the result of partial loss or lack of heme-incorporation [27]. While electrophoretically almost pure as well, the even lower R*Z* of r*Ani*UPO might be caused (at least to some extent) by the high glycosylation degree. Characteristic features of the purified proteins are summarized in Table 3.

### 3.6. Comparison of Enzyme Activities

**Figure 6:**
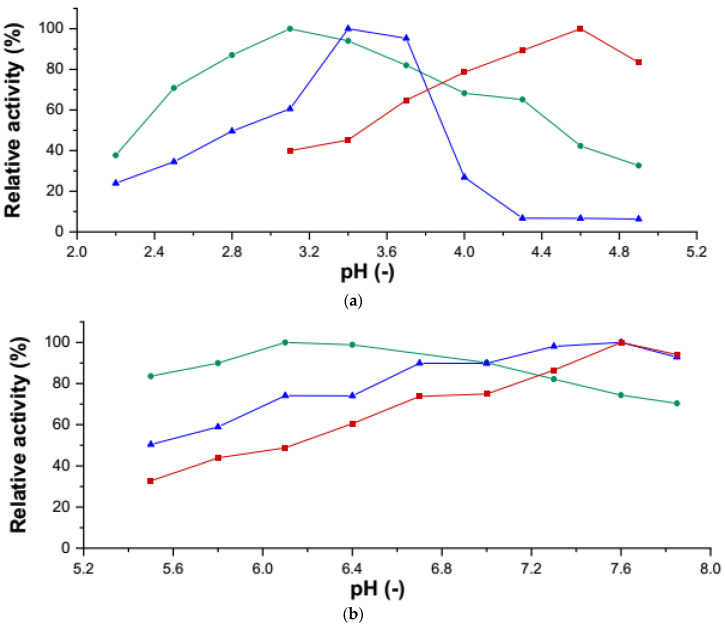
(**a**) Determination of the pH-optimum of r*Cab*UPO 1 (▲), r*Cab*UPO 2 (∎), and r*Ani*UPO (●) using ABTS as a substrate. An activity of 100% corresponds to the highest activity measured for each individual enzyme. (**b**) Determination of the pH-optimum of r*Cab*UPO 1 (▲), r*Cab*UPO 2 (∎), and r*Ani*UPO (●) using NBD as a substrate. An activity of 100% corresponds to the highest activity measured for each individual enzyme.

**Figure 7:**
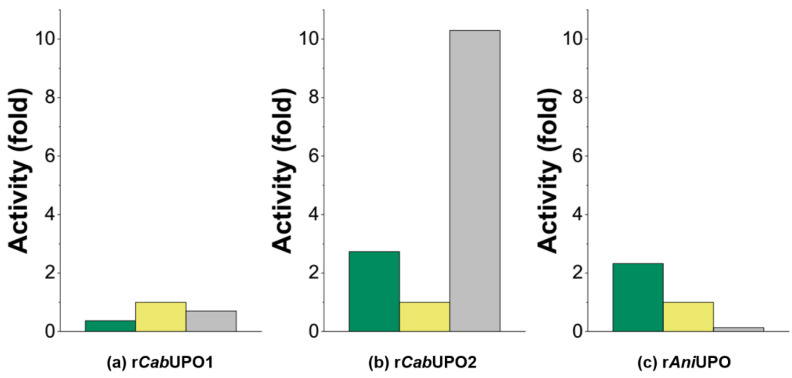
Relative activities of purified rUPO proteins towards ABTS (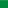), NBD (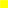), and VA (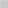). Specific activities were normalized by dividing the specific activity of a respective substrate at pH-optimum by the specific NBD activity at pH-optimum. NBD was chosen for normalization as all enzymes exhibited similar activities (10–30 U_NBD_ mg^−1^) towards this substrate (n = 1).

Purified r*Cab*UPO 1, r*Cab*UPO 2, and r*Ani*UPO were compared regarding their specific activities and pH-optima for typical UPO substrates. Storage of the enzymes at 4 °C after purification for several days led to partial inactivation, most notably in the form of irreversible protein denaturation (which became visible by precipitation). For this reason, pH-optima and activities of all samples were normalized and ‘absolute’ specific activities are not given. pH-Optima were determined with the substrates ABTS, NBD, and VA (veratryl alcohol; Figure 6). The pH-optima for ABTS oxidation varied significantly for r*Cab*UPO 1 and r*Cab*UPO 2, which is noteworthy, since both enzymes originate from the same organism. Similar behavior was observed for the evolved *Cab*UPOs [9]. Activities of r*Ani*UPO were generally more pronounced at lower pH and considerable differences in the relative activities towards the substrates investigated were noticed (Figure 7). Most notable were the differences in the VA-oxidizing activity, which was quite high for r*Cab*UPO 2, whereas *Ani*UPO showed hardly any activity towards VA. A deeper biochemical characterization of the new UPOs is out of the scope of this paper, but will be performed in the future.

**Figure 8:**
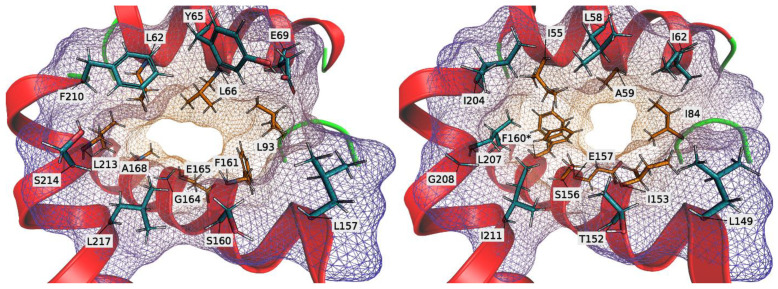
Amino acids defining the substrate access channel of r*Ani*UPO (**left**) and *Mro*UPO (**right**) in a top-down view from the protein surface to the channel bottleneck. Main-chain secondary structures are shown as cartoon helices (red) or loops (green). Side-chains (depicted as sticks) are labeled using the IUPAC standard one-letter amino acid code. The field-of-view was reduced to 60° (aids depth-perception and reduces visual cluttering). The solvent excluded surface (SES) of the channel-lining amino acids is depicted as a colored mesh (ignoring all other amino acids). The meshes are colored from blue (bottleneck distal) to orange (bottleneck proximal) and are colored in approximation as the amino-acid side-chains that either form the channel-bottleneck (in orange) or rather the outer channel perimeter (in blue). (*) F160 in the resolved structure for *Mro*UPO (5FUK) has two rotamer-solutions (while 5FUJ has two rotamer-solutions for I55 but not F160; not shown).

None of the here-studied UPO enzymes currently have an experimentally resolved tertiary structure (e.g., by x-ray diffraction, solution NMR, or electron microscopy). Their respective 3D structures were instead simulated by using the computational C-I-TASSER pipeline (version 1.0; [12]) for contact-guided protein structure prediction. Special focus was given to the visualization of the substrate access channels (Figure 8) of r*Ani*UPO and its structurally closest fit of an experimentally resolved and published UPO crystal structure (that of *Marasmius rotula* UPO/ *Mro*UPO, PDB accession: 5FUK, chain A). The 3D models of the evolved r*Cab*UPOs have recently been published [12]. The top C-I-TASSER-predicted model of r*Ani*UPO had a C-score (confidence score) of 1.98 (on a scale of –5 to +2; higher is better) and an estimated TM-score (estimated correctness of global topology) of 0.99 ± 0.04.

Comparing the simulated r*Ani*UPO model and the resolved *Mro*UPO in a structural alignment yielded a TM-score of 0.93, indicating a high global structural similarity between both proteins (based on weighted average distance of all residue pairs between the alignments). In Figure 7, this is reflected in an almost identical relative protein backbone positioning around the substrate access channel. This in turn points to individual differences in channel side-chain residues between both proteins to largely define their respective channel topology in detail (and the specific substrate spectra). *Mro*UPO’s channel-bottleneck was squarish, while r*Ani*UPO’s bottleneck was oblong. Taking Figure 8 as reference for orientation, then *Mro*UPO’s left and right bottleneck flanks were mostly defined by sidechain residues F160 and I84 and the top flank by A59. The bottom flank was defined mainly by the peptide backbone between E157 and S156. r*Ani*UPO’s channel-bottleneck was squished oblong by a much bulkier L66 that protruded into the channel from the top (A59 in *Mro*UPO), while the bottleneck was at the same time offset to the left due to F161 (I153 in *Mro*UPO). This diminished the influence of L93 in r*Ani*UPO’s bottleneck topology (I84 in *Mro*UPO). The bottom flank of r*Ani*UPO’s bottleneck was mostly defined by the peptide backbone of E165 and G164, similar to *Mro*UPO. Additionally, the left flank of r*Ani*UPO’s bottleneck was opened up relatively to that of *Mro*UPO (substituting F160 in *Mro*UPO by A168 in r*Ani*UPO). This in turn allowed r*Ani*UPO’s L62 to participate in the topology of the bottleneck flank (whereas I55 hardly did in *Mro*UPO).

To combine structural insights with measured enzyme activity, we ran docking simulations to fit VA into r*Ani*UPO’s and as comparison *Mro*UPO’s substrate access channel (Figure S7). While the simulation makes it feasible that VA fits into r*Ani*UPO’s substrate access channel, the need to present its hydroxyl-group to the heme combined with the need to accommodate its methoxy-groups makes it an awkward fit, which in turn could explain the low oxidation activity of r*Ani*UPO for VA compared with other UPOs.

## 4. Conclusions

Out of a selection of ten putative UPO genes, three enzymes exhibited activities towards UPO-typical screening substrates. These enzymes were successfully produced in *P. pastoris* in a multi-stage, high-cell-density cultivation without using an expression strategy involving first trials in *E. coli* or *S. cerevisiae*. Protein titers of 140–300 mg L^−1^ were achieved for r*Cab*UPO 1, r*Cab*UPO 2, and r*Ani*UPO, demonstrating that directed evolution is not essential for the production of this enzyme type in yeast (this result is in good agreement with [8]). Nevertheless, it is out of question that any kind of genetic engineering will have to take place in *S. cerevisiae*. Though two of the three UPOs produced originated from the same organism, all enzymes exhibited markedly different activities towards the three test substrates along with varying pH profiles. Obviously, a more thorough investigation of their activities, especially with respect to industrially relevant substrates (e.g., pharmaceuticals), will be required in the future. Nonetheless, the results presented here increase the toolbox of UPOs available for genetic engineering and heterologous expression—a general requirement for the industrial application of this enzyme type. The described expression of new UPOs, the (evolutionary) optimization of the enzymes [6,9,24,29,30,31], as well as process-engineering approaches [32,33,34,35,36,37] will certainly make UPOs a key player in technical oxy-functionalization in the future.

## Figures and Tables

**Table 1:** Investigated UPOs for recombinant expression in *P. pastoris*.

Organism of Origin, Accession Number	Individual Enzyme Designation	Calculated MW kDa w/wo Signal Peptide
*Aspergillus niger* XP_025450509	r*Ani*UPO	29.4/27.6
*Aspergillus versicolor* OJJ01936	*Ave*UPO	28.4/26.7
*Chaetomium globosum* XP_001219540	*Cgl*UPO	29.3/27.6
*Kretzschmaria deusta* ARH52644	*Kde*UPO	27.8/26.2
*Marasmius fiardii* KAF9260270	*Mfi*UPO	28.2/26.2
*Mycena galopus* Mycgal1|2083377 *	*Mga*UPO	28.6/26.4
*Candolleomyces aberdarensis* RXW18363	*Cab*UPO1	41.3/38.8
*Candolleomyces aberdarensis* RXW17618	*Cab*UPO2	41.9/39.3
*Trichoderma harzianum* XP_024770464	*Tha*UPO1	33/31.1
*Trichoderma harzianum* KKO99714	*Tha*UPO2	28.6/26.7

* ID from Mycocosm (putative allele of *M. galopus* UPO KAF8182376). MW: molecular weight.

**Table 2:** Purification of r*Cab*UPO 1, r*Cab*UPO 2, and r*Ani*UPO. Supernatant refers to culture broth clarified by centrifugation. Ultrafiltration refers to concentration of supernatant by 0.2-µm and 10-kDa ultrafiltration. HIC refers to the pooled fractions of the HIC run after concentration and dialysis against SEC-buffer. SEC refers to pooled fraction of the SEC run after concentration and dialysis against 100 mM potassium phosphate buffer (KPi) pH 7.0. Appl. refers to the volume applied to the next purification step. PF: Purification factor per purification step. Yield: Amount of enzyme activity retained per purification step. Activities were determined using the ABTS assay.

Step	Vol. Act.	Vol.	Act.	Protein	Sp. Act.	Appl.	PF	Yield
	(U mL^−1^)	(mL)	(U)	(g L^−1^)	(U mg^−1^)	%	(-)	%
**r*Cab*UPO 2**								
Supernatant	25.3 ± 1	493	12,475	-	-	100	-	-
Ultrafiltration	493.9 ± 7.8	14.8	7309	7.88	62.64	66	-	58.6
HIC	2821 ± 226	1.25	3526	37.7	74.84	80	1.19	42.7
SEC	1218.6 ± 33.6	2.2	2681	13.63	89.41	-	1.19	95
**r*Cab*UPO 1**								
Supernatant	3.7 ± 0.1	653	2435	-	-	100	-	-
Ultrafiltration	233.2 ± 1.6	7.6	1772	23.67	9.85	100	-	72.8
HIC	899.2 ± 37.6	1.2	1079	98.42	9.14	83	0.93	44.3
SEC	356.3 ± 7.9	2.8	998	21.99	16.2	-	1.77	110.9
**r*Ani*UPO**								
Supernatant	6 ± 0.3	595	3589	-	-	100	-	-
Ultrafiltration	259.6 ± 5.8	14.8	3842	6.55	39.64	60	-	107
HIC	624.9 ± 7.7	0.98	612	13.4	46.65	87	1.18	28.7
SEC	172.5 ± 1.5	1.5	259	2.34	73.57	-	1.58	48.7

**Table 3:** Size and spectroscopic characteristics of r*Cab*UPO 1, r*Cab*UPO 2, and r*Ani*UPO. Molecular mass of glycosylated and deglycosylated UPO proteins were calculated from Figure 5. Molecular weights of the mature proteins, i.e., excluding the N-terminal secretion signal, were predicted using SignalP 5.0 [28].

Feature		r*Cab*UPO 1	r*Cab*UPO 2	r*Ani*UPO
M_W_ glycosylated	(kDa)	45	50	60
M_W_ deglycosylated	(kDa)	39	42	28
M_W_ predicted	(kDa)	38.8	39.3	27.6
Soret region	(nm)	417	419	420
Charge transfer band 1	(nm)	570	572	569
Charge transfer band 2	(nm)	535	540	541
Reinheitszahl	(A_Soret_/A_280_)	2.5	1.5	1.2

## Data Availability

Data is contained within the article or supplementary material.

## Supplementary Materials

The following supporting information can be downloaded at: https://www.mdpi.com/article/10.3390/antiox11020223/s1 Figure S1: Screening of supernatant of *P. pastoris* clones transformed with UPO expression plasmids; Figure S2: Production of recombinant UPOs with *P. pastoris* X33 in a bioreactor; Figure S3: Purification of UPOs by hydrophobic interaction chromatography (HIC); Figure S4: SDS-PAGE analysis of pooled fractions after HIC purification, Figure S5: Purification of UPOs by size exclusion chromatography; Figure S6: Absorption spectra of purified proteins; Figure S7: VA-docking simulation results for AniUPO and MroUPO; Table S1: Primers used for the construction of pPpB1-based UPO expression plasmids; DNA-Sequences of UPO genes.
