# Supplementary material for: Broadening the Biocatalytic Toolbox—Screening and Expression of New Unspecific Peroxygenases
Source: Antioxidants (Basel). 2022 Jan 24;11(2):223. doi: 10.3390/antiox11020223 (PMC8868357; doi:10.3390/antiox11020223)
Supplement: Supplementary file 1 [file antioxidants-11-00223-s001.zip › antioxidants-1498066-supplementary.pdf]

# Supplementary Information

# Broadening the biocatalytic toolbox – Screening and expression of new unspecific peroxygenases

Sebastian Bormann[1], Harald Kellner[2], Johanna Hermes[1], Robert Herzog[2], René Ulrich[2], Christiane Liers[2], Roland Ulber[4], Martin Hofrichter[2], Dirk Holtmann[1,3, 5]

[1] Industrial Biotechnology, DECHEMA Research Institute, Theodor-Heuss-Allee 25, 60486 Frankfurt am Main, Germany

[2] Technische Universität Dresden, International Institute Zittau, Chair of Environmental Biotechnology, Markt 23, 02763 Zittau, Germany

[3] Institute of Bioprocess Engineering and Pharmaceutical Technology, Technische Hochschule Mittelhessen, Wiesenstraße 14, 35390 Gießen, Germany

[4] Institute of Bioprocess Engineering, University of Kaiserslautern, Gottlieb-Daimler-Strasse 49, 67663, Kaiserslautern, Germany

[5] Fraunhofer Institute for Molecular Biology and Applied Ecology (IME), Research Division Bioresources, Ohlebergsweg 12, 35392, Giessen, Germany

# Supplementary Results

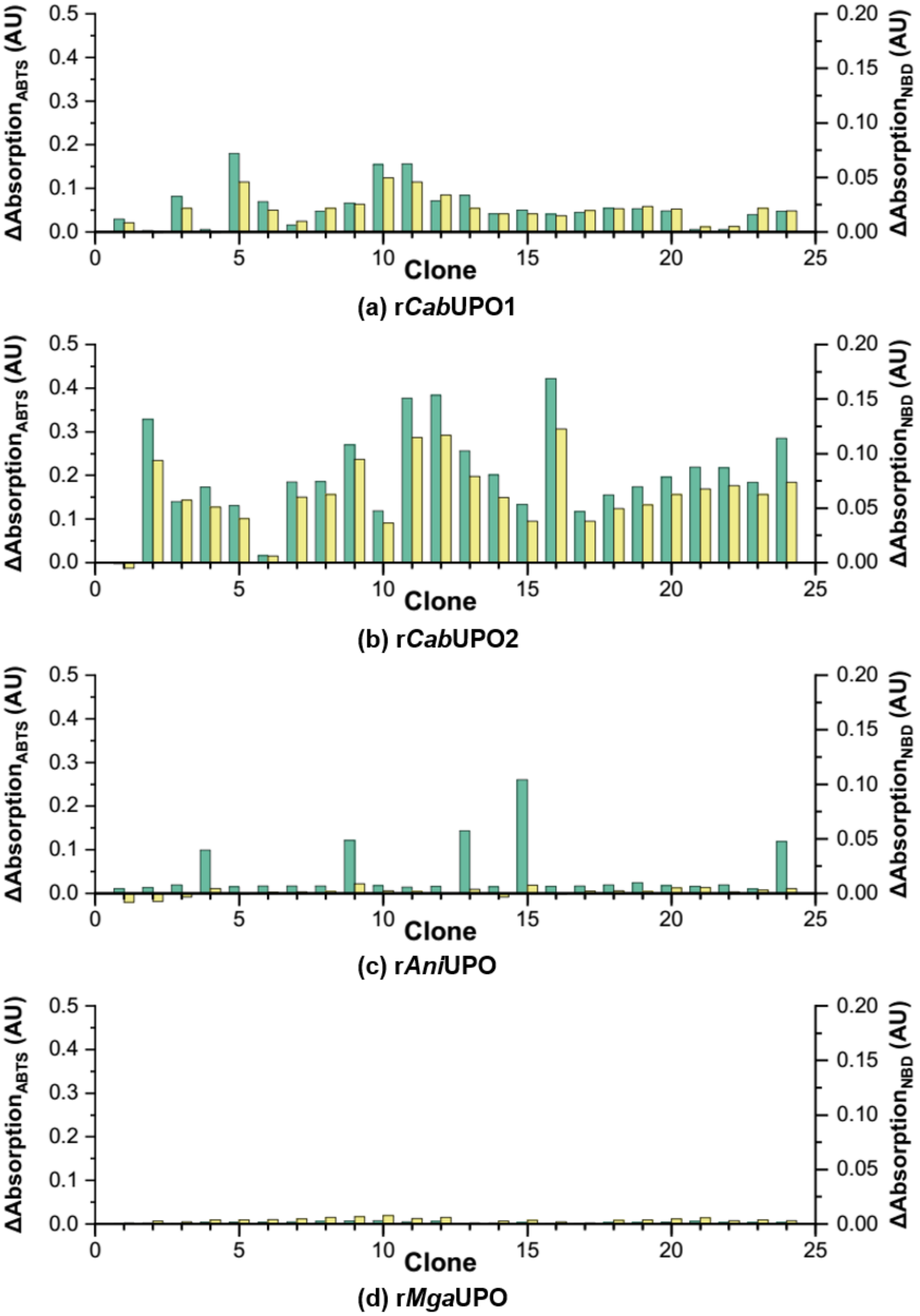


SI Figure S1: Screening of supernatant of *P. pastoris* clones transformed with UPO expression plasmids for activity using ABTS (■) and NBD (■) assays after cultivation in deep well plates for 94 h. The activity was determined by means of an endpoint measurement after 30 min of reaction time. r*Mga*UPO (d) exemplarily depicts a screening yielding no active clones. Clones chosen for subsequent larger scale production were: r*Cab*UPO 1, clone 11, r*Cab*UPO 2, clone 16, r*Ani*UPO, clone 15.

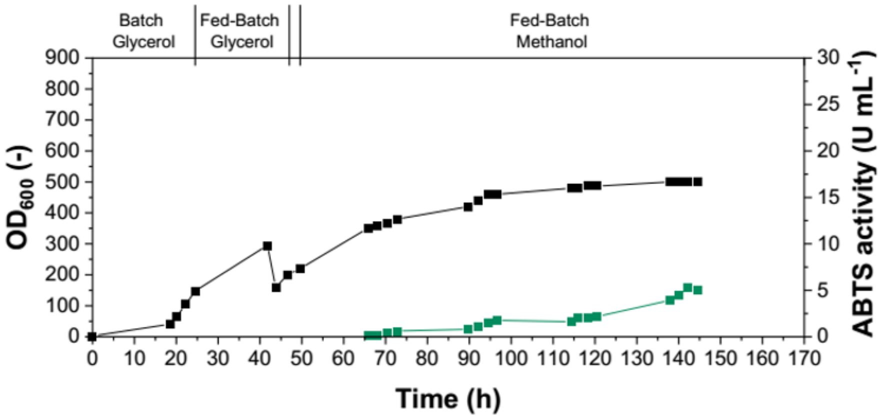

**(a) r*Cab*UPO1**

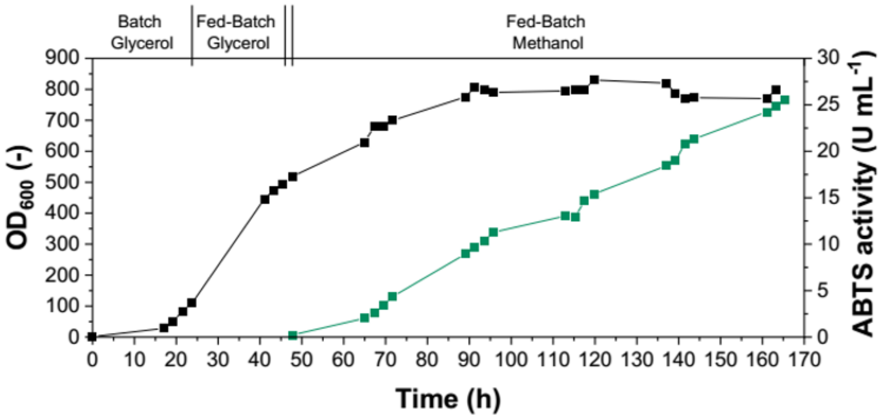

**(b) r*Cab*UPO2**

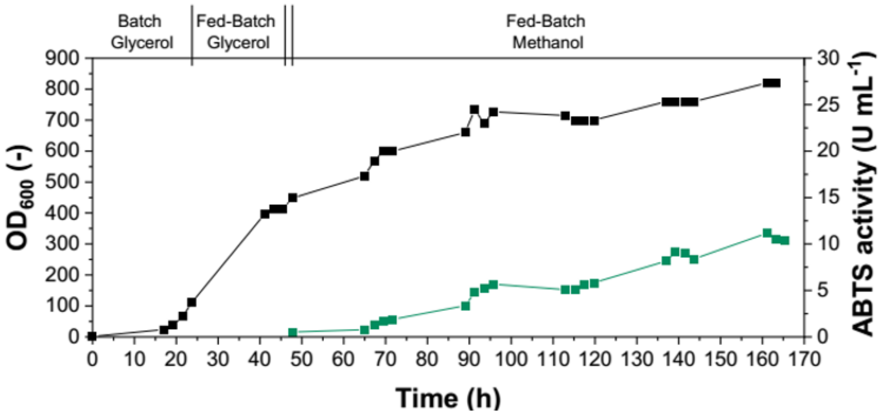

**(c) r*Ani*UPO**

SI Figure S2: Production of recombinant UPOs with *P. pastoris* X33 in a bioreactor. Phases of the glycerol and methanol (fed)-batch process are indicated. A double bar marks the starvation phase preceding the methanol fed-batch. Cultivation was monitored by measurement of $OD_{600}$ (■) and enzyme production by determination of the ABTS activity (■). The drop in OD at 40 h for r*Cab*UPO 1 was the result of losses of culture broth due to overpressure in the reactor. The loss of culture broth was compensated by aseptical addition of fresh medium, which lead to this drop in cell density.

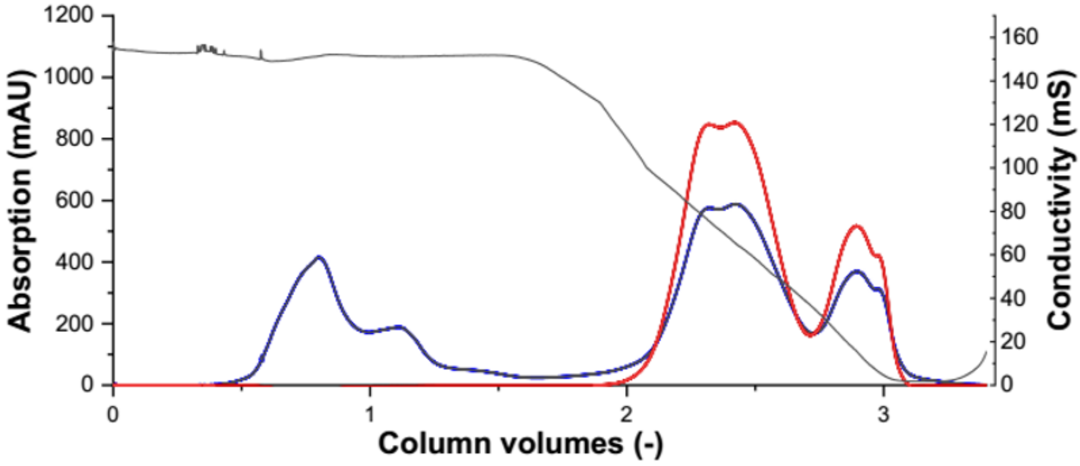


(a) r*Cab*UPO1

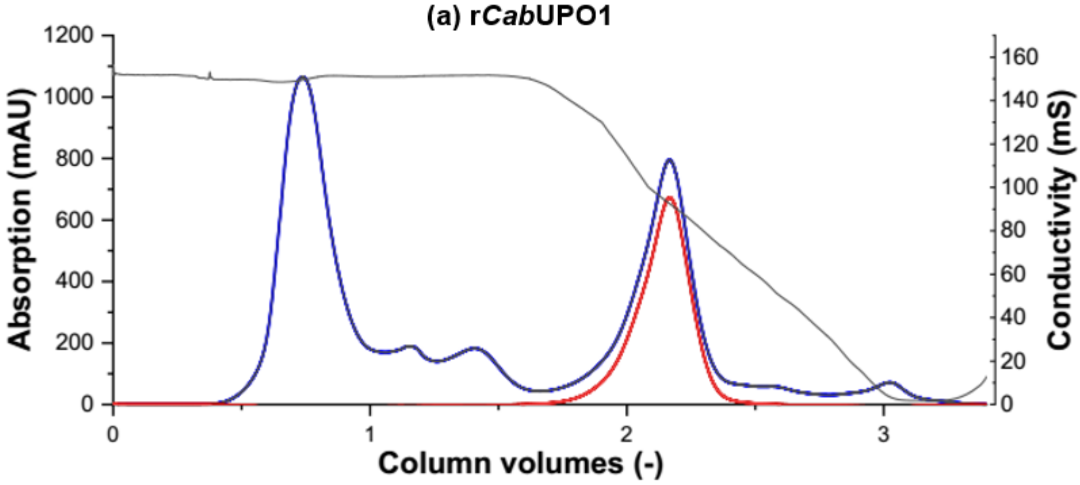


(b) r*Cab*UPO2

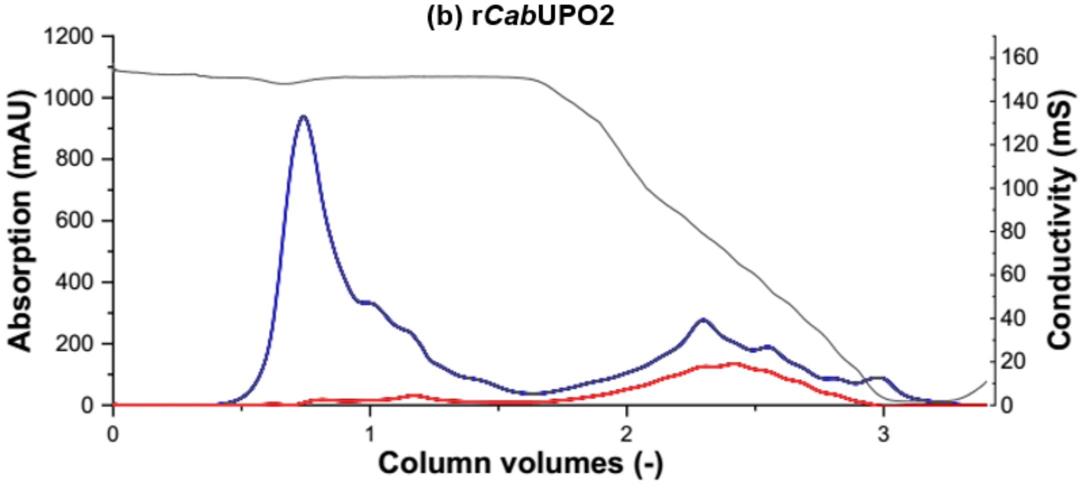


(c) r*Ani*UPO

SI Figure S3: Purification of UPOs by hydrophobic interaction chromatography (HIC) using an ammonium sulfate gradient from 1 M to 0 M as indicated by the reduction in conductivity (—). Absorption was determined at 280 nm (—) and heme-specific absorption at 420 nm (—).

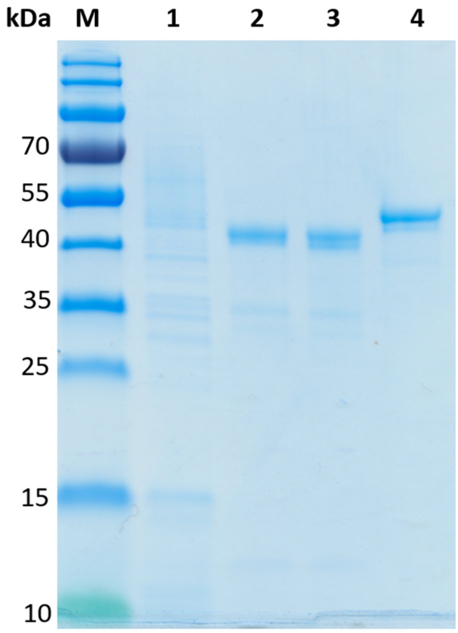


SI Figure S4: SDS-PAGE analysis of pooled fractions after HIC purification. Lanes: **M** PageRuler Prestained (ThermoFisher), 1 rAniUPO, **2** r*Cab*UPO 1 peak 2.4 CV,**3** r*Cab*UPO 1 peak 2.9 CV,**4** r*Cab*UPO 2

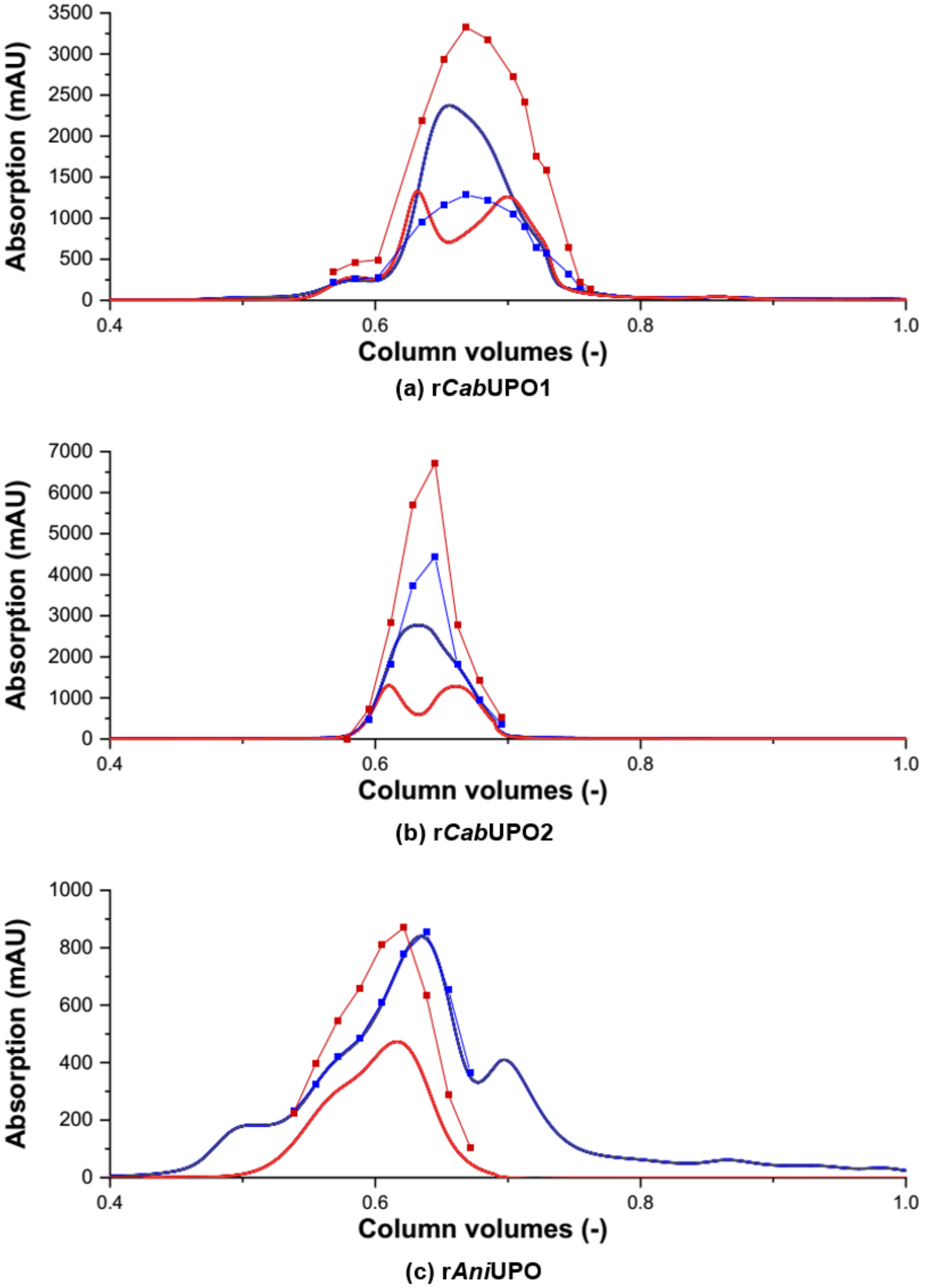


SI Figure S5: Purification of UPOs by size exclusion chromatography. Absorption was determined online (280 nm —, 420 nm —) and offline (280 nm ■, 420 nm ■).

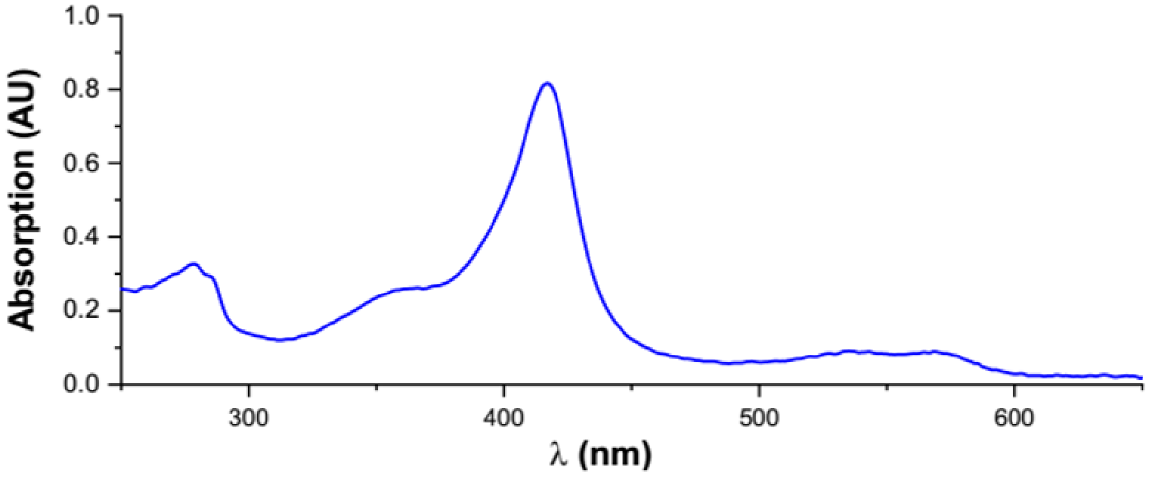


(a) r*Cab*UPO1

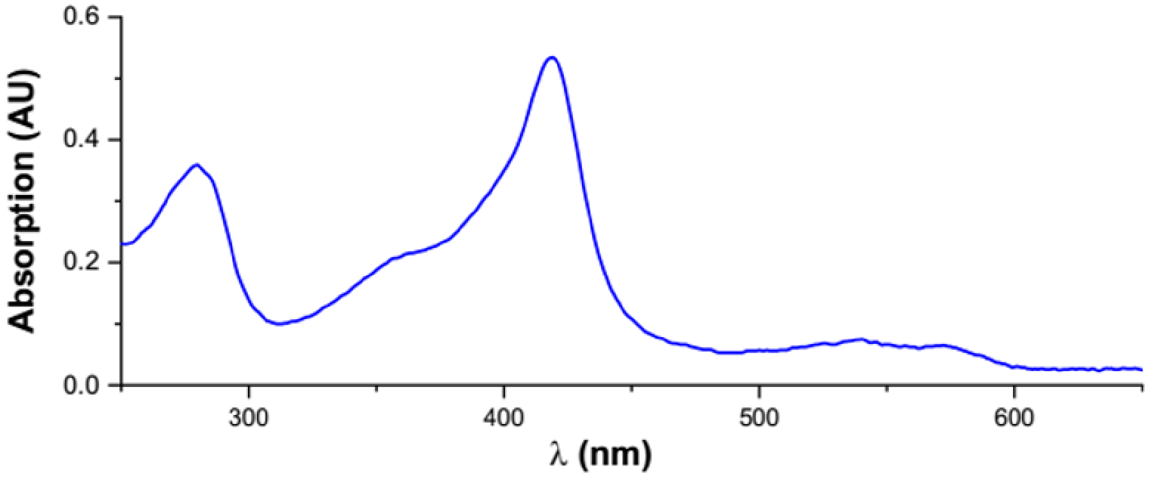


(b) r*Cab*UPO2

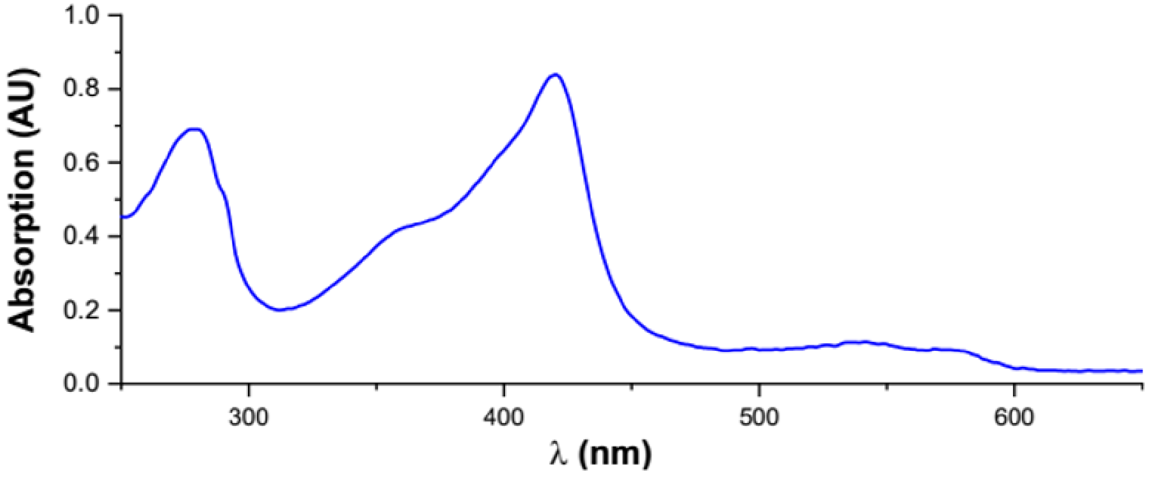


(c) r*Ani*UPO

SI Figure S6: Absorption spectra of purified proteins.

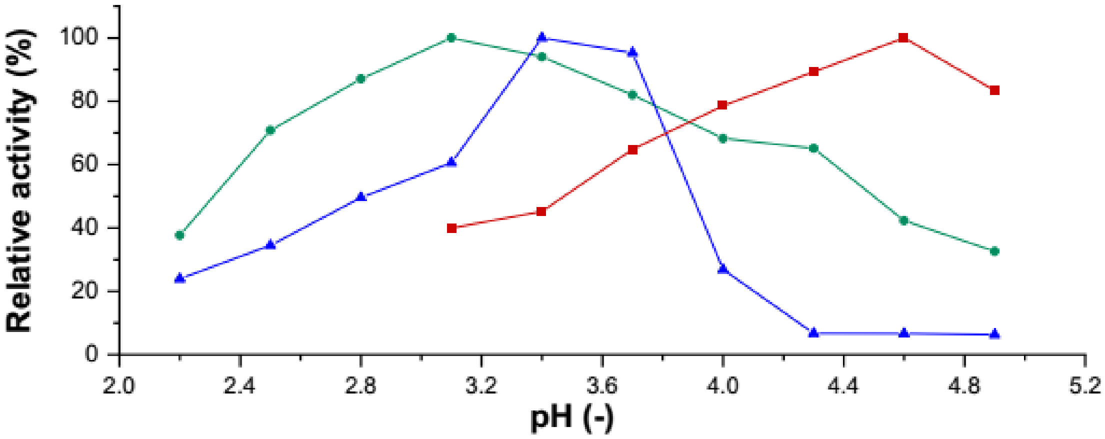

SI Figure S7: Determination of the pH-optimum of r*Cab*UPO 1 (▲), r*Cab*UPO 2 (■) and r*Ani*UPO (●) using ABTS as a substrate. An activity of 100% corresponds to the highest activity measured for each individual enzyme.

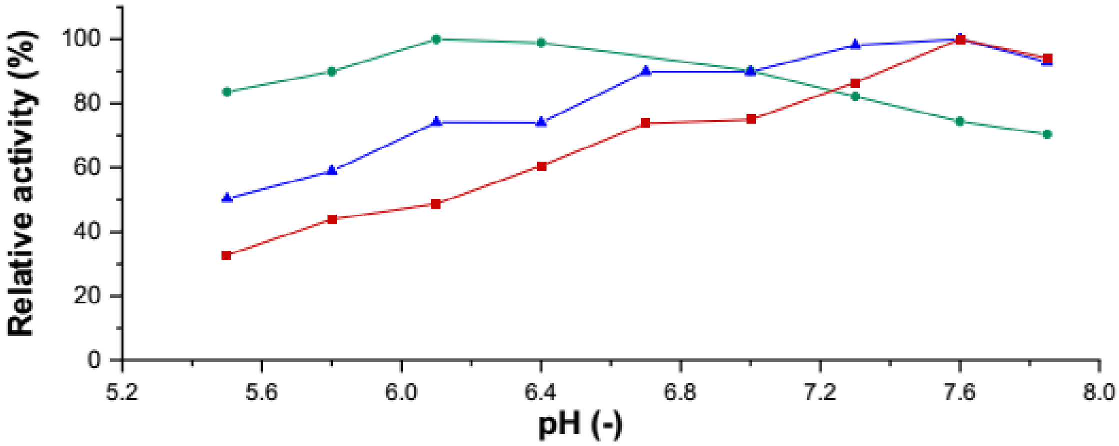

SI Figure S8: Determination of the pH-optimum of r*Cab*UPO 1 (▲), r*Cab*UPO 2 (■) and r*Ani*UPO (●) using NBD as a substrate. An activity of 100% corresponds to the highest activity measured for each individual enzyme.

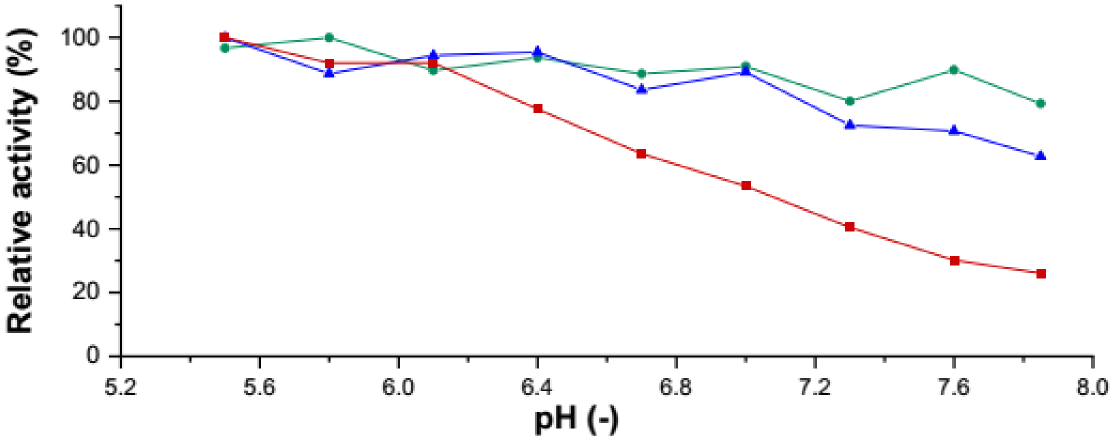

SI Figure S9: Determination of the pH-optimum of r*Cab*UPO 1 (▲), r*Cab*UPO 2 (■) and r*Ani*UPO (●) using veratryl alcohol as a substrate. An activity of 100% corresponds to the highest activity measured for each individual enzyme.

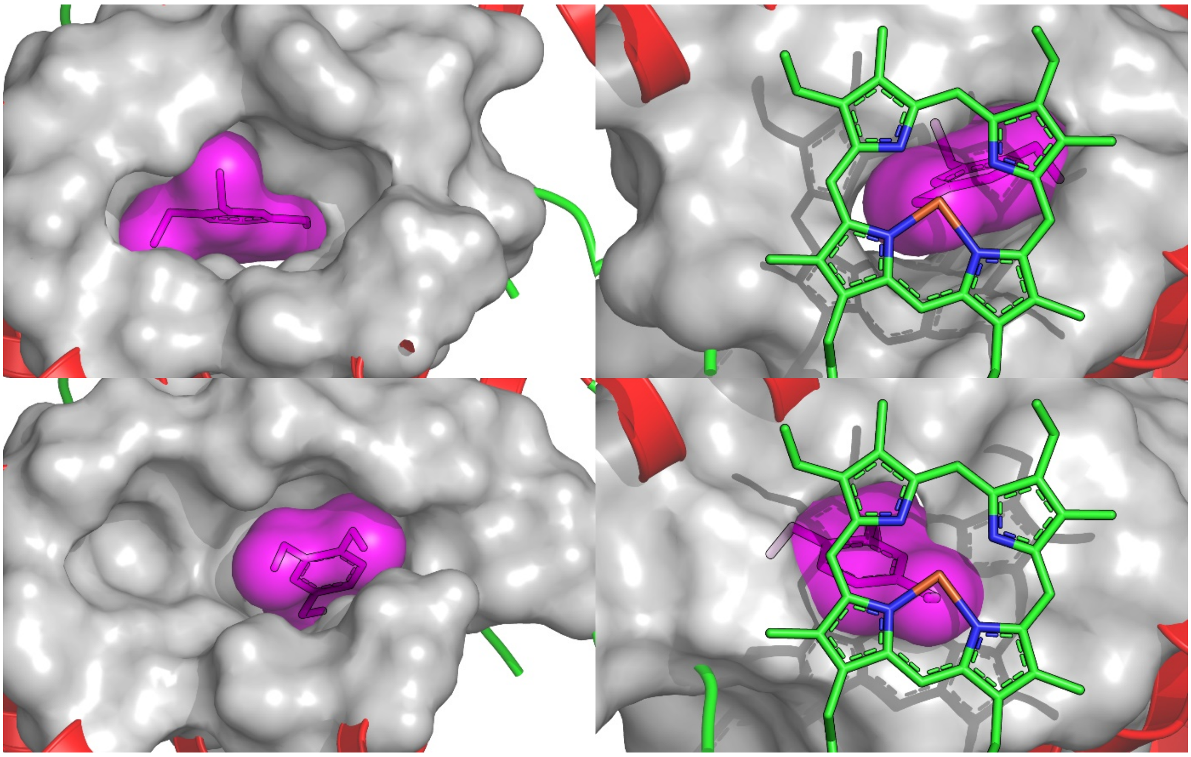

SI Figure S10: VA-dockingsimulation results for *Ani*UPO (upper pictures) and *Mro*UPO (lower pictures). Each picture on the left is a top-down view from the proteins surface into the substrate access channel. Pictures to the right are the respective bottom-up view. VA in magenta as sticks surrounded by its approximated SES. Heme as sticks; carbon = green, nitrogen = blue, iron = brown.

SI Table S1: Primers used for the construction of pPpB1-based UPO expression plasmids.

| **Target** | **Primer (5'-33)** | **TM °C** |
|---|---|---|
| pPpB1 | tcaagaggatgtcagaatgc<br>CGTTTCGaataattagttgttttttgatcttctcaag | 60 |
| AniUPO | aagatcaaaaaacaactaattattCGAAACGATGaagaccaccaccc<br>ggcattctgacatcctcttgaTTAgccacgccagga | 62 |
| AveUPO | aagatcaaaaaacaactaattattCGAAACGATGaagacccctatcct<br>ggcattctgacatcctcttgaTTAgttatcggaatccttgac | 59 |
| CglUPO | aagatcaaaaaacaactaattattCGAAACGATGcgcaccagcctg<br>ggcattctgacatcctcttgaTTAgaagccgaagtggg | 62 |
| KdeUPO | aagatcaaaaaacaactaattattCGAAACGATGaagttcgccaccc<br>ggcattctgacatcctcttgaTTAgccgaagatctcgc | 62 |
| MfiUPO | aagatcaaaaaacaactaattattCGAAACGATGaagaacctcctctcc<br>ggcattctgacatcctcttgaTTAcacatcagggagctg | 61 |
| MgaUPO | aagatcaaaaaacaactaattattCGAAACGATGcagctcaagctga<br>ggcattctgacatcctcttgaTTAatggaagacgatgccg | 62 |
| CabC27 | aagatcaaaaaacaactaattattCGAAACGATGatcagccagagcttc<br>ggcattctgacatcctcttgaTTAcagctggccgtag | 62 |
| CabC32 | aagatcaaaaaacaactaattattCGAAACGATGgtgagcaagccc<br>ggcattctgacatcctcttgaTTAcagctggccgtaag | 62 |
| ThaUPO1 | aagatcaaaaaacaactaattattCGAAACGATGaagtacctgctcagc<br>ggcattctgacatcctcttgaTTAggcgccgatctg | 62 |
| ThaUPO2 | aagatcaaaaaacaactaattattCGAAACGATGctgctgtccaac<br>ggcattctgacatcctcttgaTTAatcctcggagaacttctg | 60 |

DNA-Sequences of UPO genes

**UPO from Aspergillus niger (AniUPO)**

ATGaagaccaccaccctcctgtgcctcgccgccgctctcacccaggctaacgccttccctcagcagggcgc
ccctcatcctctcccttggtcccccccggccctaacgatgtgcgcgctccttgccccatgctgaacaccc
tggccaaccatggctacctgccccacaacggcaaggacatcaccgagcagcacaccatcaacgccctgtac
aacgccctcggcatcgaggaggagctcgccgtctacctgcaccaggaggccgtgaccaccaaccccgcccc
taacgccaccaccttctccctcaacgatctgagccgccatgacatcctggagcatgatgcctccctgtccc
gccaggacgccttcttcggcgataaccatgacttcaaccagaccatcttcgatgagacccgctcctactgg
accagccccatcatcgacgtcaagcaggccgccctgagccgccaggctcgtgttaacaccagcatggccac
caaccccaactacaccatgtccgagctgggcaactccttctcctacggcgagaccgccgcctacatcatcg
tcctcggcgacaaggagaagggcctcgtgaaccgctcccgcgtggagtacctgttcgagaacgagcgcctg
cctctcgacctgggctggtcccgtgccaaggagaacatcaccttcgacgacctgtccaccatgctgcagcg
catcatcaacgccaccggcggcgagtccgagttcgaccgtgagctggccaagcgcggcggcgttcatgtgg
gctcctggcgtggcTAA

**UPO from Aspergillus versicolor (AveUPO)**

ATGaagacccctatcctgtccctggccgtgggcctggccggttgtgctgtcgcttaccctgagtacctcga
gggccgcagcgagtggcaggcccaagagtggatcgcccctggccctgacgattcccgcggcccttgccctg
gcctcaacaccctggccaaccatggctacctgcctcgcaacggcaccggcatcaccctcgacatcctgaag
gatgccatgctcgagggcttcaacatcgagcatagcgatgccgagctcctgttcacccaggccatccgcac
ctcccctaactggcctctcaccctcagcttcgacctcgccgatctgggccgccatggcatcctcgagcatg
atatctccctctcccgctccgacgcctacttcgccgatcctaaccccttcaacgagaccgtctgggccgag
accgccagctacttcaccacccccatgatcaccgtggaccagctggccaaggcccgcatgggccgtctcgc
tacctccaagaagtccaaccccgagttcgagctgagctggctggccgatggcttcagctggggcgagtgcg
ccagcttcttcgagatcatggccgatggcaccaccggcaccgtcgataagaagtacatcgagtactggttc
cgcaacgagcgcatgcctaccgagatcggctggcagcgccgcaacaccaccatgcagggcagcgagcgccg
caagtactccaacctgctgcaggaggccgccggcgtcaaggattccgataacTAA

**UPO from Chaetomium globosum (CglUPO)**

ATGcgcaccagcctgctccccgccctggctgctgtgtcccctgtgctggccggcttcgatacctgggcccc
ccctggtccctacgacgtccgtggtccctgccccatgctgaacaccctcaccaaccatggcttcttccctc
acgatggccaggatatcgaccgcgagaccaccgagaacgccctgttcgatgccctccatgtcaacaagacc
ctggccagcttcctgttcgacttcgccctgaccaccaaccctatcgccaacagcaccaccttcagcctgaa
cgacctgggcaaccataacgtcctggagcacgacgcctccctgagccgcgctgacgcttaccacggcagcg
tcctggccttcaaccacaccatcttcgaggagaccaagtcctactggaccgatgagaccgtgaccctcaag
atggccgccgatgcccgctactaccgcatcaagagctcccaggccaccaaccctacctaccagatgagcga
gctcggcgacgccttcacctacggcgagtccgccgcctacgtcgtcctcttcggcgacaaggagagccaga
ccgtgccccgctcctgggtcgagtggctgttcgagaaggagcagctgccccagcacctgggctggaagcgc
cctgctacctccttcgagctgaacgatctggataagttcatggccctcatccagaactacacccaggagat
cgaggagccctcctgcgagagccgcaagcagcgccgtaagcctcgcggcccttcccacttcggcttcTAA

**UPO from Kretzschmaria deusta (KdeUPO)**

ATGaagttcgccaccctcctcttccccgccgtcgtcctcggcgctgcttgcccttacggcaccttctcccc
tgagaagcccaccgacacccgcggcgtgtgccctatgctgaacgccctggccaaccatggcttcctccctc
gcgatggccgcgacatcaccgagaaccagaccgtgaccgccctcaacgacgccctgaacctcacccctgac
ttcggccagttcctcttcaccgccgcccgcctctccaaccctaagcctaacgccaccaccttcaacctcga

tcacctggaccgccacgacctcttcgagcatgacggcagcctgagccgccaggacgcctacttcggccagt
ggtcccgcttcaacgccaccgtgtggaactggaccatgcagtactacaccggcgatgtgctcgacgtccag
atgatcgccaacagccgcgcccagcgcgccatgagaagcaacctgaccaaccccgagtacaagctgtccgt
cgtgggctacgacttcagcgtcgccgagaacgccgccatcctcagcatcctcggcgataaggtcacccaga
cctgccctaaggtgttcgccgattacctcttctccaaggaggagctgccctactccgtcggctggaagaag
tccgagctccctatctccctgcaggatctgatcaccaccttccacgacatcgagctggccaccgccttccc
cgcccctcctcctcctgacaactccggcgagatcttcggcTAA

**UPO from Marasmius fiardii (MfiUPO)**

ATGaagaacctcctctccctgatcttcatcgccgtcgccgtcaccgcctccaccgactctcaggtggactg
gtccagccaccgctggcaggcccctggtccttccgatacccgcggcccttgccccggtctcaacaccctgg
ccaaccatggcttcctccctcgcaacggccgcaacatctccatccccatgatcctgcaggccggccatgag
ggctaccacatcgagccctccatcctgaccatcgccgccaaggtgggcctgctgaccagccctagcgatca
gcccaccaccttctccctcgaggacatcaaggcccacggcgtcatcgagcatgatgccagcctctcccgcc
aggatatcgccctgggcgataacgtccacttcaacgagaccatcttcagcaccctggtcaacagcaaccct
cgctccgatgtgtacaacaccaccagcgccggccaggtcctccaggctcgtctggctgatagcctgaagaa
caaccctaacgtgaccaacaccgatctgaccgatgtcatccgcggcgccgagagcgccttctacctctccg
tcatgggcgaccctatccagggcgtcgcccctaagaagttcgtgcagatcttcttccgcgaggagcgcctg
cccatcgaggagggttggaagcgcaccaacatcagcatcaccctcaacgtcgtcggcgccctcgtcgataa
gaccttcgaggccgatcctgcctgggagcctaccggcaacaactgccctggcatccagctccctgatgtgT
AA

**UPO from Mycena galopus (MgaUPO)**

ATGcagctcaagctgagctccctgatcgtcgccagcgtcgtgctcgtcggcaaggtcaacgcctacgccgc
ccccgctggccatgagttcatcgcccccaccagcaccgaccagcgctccccttgccccggtctcaacaccc
tcgccaaccatggctacctgcctcgcaccggcgccaacttcaccgtgacccagatcatggatgccgccctc
gccggcttcaacgtcaactgggatgccatcctcgtggccgccaagttcggcctgctcagcggcgctgacgc
caccaccttcgagaccatgtccctcggcccctggccctgcacaacctcgtcgagcacgacgcctccatct
cccgcaacgacttcggccccgacggcaccggtgacaacgtgcacttcaacgagaccaccttctccaccctg
gccaacgccaaccccggcaaggattactacgatcctgtcagcgccggcgccgtccagcacgctagactggc
tcactccgtcgccaccaaccctaacgtcaccaacacccagaaggagttcaagctgcgcagccgcgagtccg
ccctgtacctcagcatcttcggcgacccctcaccggcatcgccagcaaggagttcgtgcagatcttcttc
cgcgaggagcgcctccccttcgccgagggttggtccaagcccaccaccctgatcgacagctccaccctcga
tcccatcggcaacatcatccagaacgccagcgactggaccgagacccagtcctgcgagcctctcgtcctcg
gctccggcatcgtcttccatTAA

**UPO 1 from Candolleomyces aberdarensis (CabUPO 1)**

ATGatcagccagagcttcgtgtccctgaccgtgctgctgctcggcctcgtcaacctgagccccgccttcgc
cttcccccagtacggctccctggccggtctctccgcccgtgatctgaacgtcctcatccctcgcctgaacg
aggtcgatcctcccacccctcccggccctctcgcttacaacggcaccaagctcgtccatgacgatgcccac
cccttcaaggcccccgagcagggtgatatccgcggcccttgccccggcctgaacaccttggccaaccatgg
ctacctccctcataacggcgtcgccacccctagccagatcatcgaggccgtccaggagggcttcaacatgg
agcatgccaccgccctgttcgtcacctacgccgcccacctcgtggacggcaacctcgtgaccgatctgctc
tccatcggcgagaagaccggcctgaccggcctggatcctcccgctcctgccatcgtgggcggcttgaacac
ccatgccgtcttcgagggcgacgcctccatgacccgcgccgacttcttcttcggcgataaccataacttca
accagaccctgttcgaccagttcgtggacttcagcaaccgcttcggcggcggcttctacaactacaccgtg
gccgccgagctgcgcttccagcgtatccaggagagcatcgccaccaaccctcagttctccttcatctcccc
ccgcttcttcaccgcctacgccgagagcaccttccctgtcaacttcttcgtggacggccgcagcaccgaga

agaagctcgacatggaggccgccacctccttcatccgcgacggcaagtaccctcaggacttccatcgcgcc
gcccagccttcctccaccgagggtatcgatatcgtggcctccgcccaccctgtggcccctggtgaaaaccg
cgacggcaaaatcaacaactacgtccccgaccctacctccgccgacttctccaccttctgcctgctgtaca
ccaacttcgtgaaccagaccatcggcggcctctaccctaaccccaccggcgtgctccgccgcaacctgatc
aagaacctccgcttcttctacagcggcatcgccgatgccggctgcgaggagctcttcccctacggccagct
gTAA

**UPO 2 from Candolleomyces aberdarensis (CabUPO 2)**

ATGgtgagcaagcccttcgccctcctgaccacctacctgctgatcctgggcaccctctccacctccctcgc
cttccccggcctccaggtccctaaccgcgccttgctgcgccgccaggagggtggtggttcccagggtggcg
gcagctccgagcagggtgtggaccctcctcctcccggccctccttccttcaccggcaccaagctcgtc
aacgatgccgatcacccttggcagcccctgcgcgagggcgatatccgcggtccttgccccggcctcaacac
cctggccagccacggttacctgccccgcgacggtgtcgccacccctgctcaaatcatcaccgccacccagg
agggcttcaacttcgagaacaacgccgccatcgtcgccacctacctgggccatctcctcaacggcaacctg
gtgaccgatctgctgtccatcggcggcgccaccccctaagaccggccctcctcctccgcccccctgctcatgc
tggcggtctcaacgtgcatggcaccttcgagggcgacgccggcatgacccgcgctgatgagttcttcggcg
acaaccacagcttcaaccagaccctgttcgacaagttcgtggacttcagcaaccgctacggcggcggcttc
tacaacctcaccgtcgccggcgagctccgctacagccgtatccaggacagcatcgccaccaaccctgagtt
ccagttcaagaacgtgcgcttcatcaccgcctacggcgagaccgtcttccctatcaacctgttcgtggatg
gccgcgtgaccaccgaccgcaagctgtccatggaggatgccgccagcatcttccgcgatatgcgcttccct
gatgacttccaccgctccgccgtccctgcctccaacgagggtgccgaccaggtcctcgccgcccatccttg
ggtgcctggcggtaacgccgacaaccaggtgaacaactacgtcgaggatcctgacagcgccgacttcaccc
acctctgccgcctgtacgagttcgtggtgggctccgtgcaggagctctaccccaaccctaccggcatcctg
cgccgcaacctgatcaagaacctccattactggtggaccggcgtcaacgtcgccttcggcggctgcgatga
gctcttcccttacggccagctgTAA

**UPO 1 from Trichoderma harzianum (ThaUPO 1)**

ATGaagtacctgctcagcctggtcgccatcagcaccctgagcaacatcggcgccgcccagctgctggatct
ccctcctctcttcgatccctacgatcctaagttcgacgactggcagcccgccggccctgacgatagccgtg
gtccttgccctggcctgaacagcctggccaaccacggcttcctcccccatagcggcaagaacatcaccatc
atcgatctcctccgcggcgcctacctgggcctcggtgttagccctgagatctccatcgtggtgggcgccgc
cgagctcatcaagagctaccgcctcgccgccttcgatctccacgagctctccaaccacggcttcatcgatc
atgattgctccctctcccgcgccgacatcggcgacggtaacaacaacgacttcaacgagaccatctggagc
gtgcctctggaggtcctgatgaactacaccatcatcacccctgaggccatcggcgccgctcgctctgctcg
tgacctgttcgacatcgcccacaaccctaaccagcagtgcggcgcccgctccatcgccatcggtgctctcg
agaacggcctgctcatccagtccctgggcggcaagcctgagctcgagtgggtccgctccatcttcgagcag
cagcgcatccctaccaacctgggcttccgccccaagcccatcgtcctcaacaacgccgtgggcgtcgccac
cgtcggcttggaaagcctgctgtcccagcccaacctgaccgatctcctgggcagcgtcgtgatcaagaccc
ctgccgacctgctggccgaggtgttccctgtcaaggagtacaacctcacctacatcctcgaggtcctgggc
ctggccggcttccctgctgtggatctgtccttcctcaccggctacaagaaggccatccagatcggcgccTA
A

**UPO 2 from Trichoderma harzianum (ThaUPO 2)**

ATGctgctgtccaacgtcttctccttcgccgtgttcgccaccgccgcctacgcctctgtgcaggtccagca
gtggatgcctcccggccccgacgatagccgcggtccttgtcccatgctcaacaccctcgccaaccatggct
acctcccccactccggccgccatatcagcccttcccagatcgaggatgccttcaccaacttcctcaacatc
gaggccggcttcgccgccgccgctaaggacttcgccaaggcctggggcaacgataccttcgatctcgatga
tctgaacacccctgatatcctccagcacatcgccagcctgacccgcgacgactacacctcccgcaaccctc

acctcaagcctaacttcctccgcatcgagcgcctgatgtccgatagccctacctccttcatcgacgtcgac
tccatcgccaagacccgcctccgcgtcgagagcgagtccacccctaacgccctctccaagaaccagatgaa
cgccgccctcttcgaggccgccatggtgctggtcatgatgtccgaccactcccctgacgccgagttcaacc
ccccccctgtccgcctaccagggccctaaggatcgcatccgcgcctggttcgagcaggagcgcttccctacc
gagttcggctggcagcctagcgcccgcaccatcaagctcgccgatctgaaccccgccatcaacggcatcat
caagtccatggagcgccaggacaagagcggccgcgtcgctcagaagttctccgaggatTAA
